# Ribosome-Inactivating Proteins from Plants: A Historical Overview

**DOI:** 10.3390/molecules21121627

**Published:** 2016-11-26

**Authors:** Andrea Bolognesi, Massimo Bortolotti, Stefania Maiello, Maria Giulia Battelli, Letizia Polito

**Affiliations:** Department of Experimental, Diagnostic and Specialty Medicine-DIMES, Alma Mater Studiorum, University of Bologna, Via San Giacomo 14, 40126 Bologna, Italy; andrea.bolognesi@unibo.it (A.B.); massimo.bortolotti2@unibo.it (M.B.); stefania.maiello2@unibo.it (S.M.); letizia.polito@unibo.it (L.P.)

**Keywords:** anticancer drugs, antiviral proteins, immunotoxins, plant lectins, plant toxins, ribosome-inactivating proteins, ricin, science history

## Abstract

This review provides a historical overview of the research on plant ribosome-inactivating proteins (RIPs), starting from the first studies at the end of eighteenth century involving the purification of abrin and ricin, as well as the immunological experiments of Paul Erlich. Interest in these plant toxins was revived in 1970 by the observation of their anticancer activity, which has given rise to a large amount of research contributing to the development of various scientific fields. Biochemistry analyses succeeded in identifying the enzymatic activity of RIPs and allowed for a better understanding of the ribosomal machinery. Studies on RIP/cell interactions were able to detail the endocytosis and intracellular routing of ricin, thus increasing our knowledge of how cells handle exogenous proteins. The identification of new RIPs and the finding that most RIPs are single-chain polypeptides, together with their genetic sequencing, has aided in the development of new phylogenetic theories. Overall, the biological properties of these proteins, including their abortifacient, anticancer, antiviral and neurotoxic activities, suggest that RIPs could be utilized in agriculture and in many biomedical fields, including clinical drug development.

## 1. The First Studies

A series of plants used from ancient times for medicinal purposes in various parts of the world (reviewed in [1]) produce proteins that are today referred to as ribosome-inactivating proteins (RIPs) (reviewed in [2]). Some of these RIP-expressing medicinal plants are very toxic, and their toxicity has been known since antiquity (reviewed in [3]).

The history of RIPs began when the interest of the scientific community turned to the toxicity of plant poisons, in particular those from the seeds of *Ricinus communis* L. (castor bean) and *Abrus precatorius* L. (jequirity bean). In both cases, the toxins were identified at the end of nineteenth century to be proteins [4,5]. They were partially purified at the University of Dorpat (now Tartu in Estonia) and named ricin [6] and abrin [7], respectively. These studies, performed for a doctoral thesis, identified ricin and abrin as haemagglutinins, and their toxicity was erroneously attributed to their ability to agglutinate red blood cells.

The next stage of RIP history involved the pioneering immunological research of Paul Ehrlich at the Institute of Infectious Diseases in Berlin. He observed protective effects from the toxicity of either abrin or ricin in mice by feeding them low amounts of the toxins. The immunized animals still conserved sensitivity to the other toxin, demonstrating the specificity of the immunity. He found that the serum of the immunized animals contained proteins that were able to precipitate the specific immunizing toxin but not the other toxin. He also found that the anti-toxin immunity could be transferred from the mother to the offspring both through blood during pregnancy and through milk during the breast feeding [8,9].

The enzymatic nature of ricin action was suggested early on [10]. Afterwards, attention was focused on the role of haemoagglutination activity in the toxicity of castor bean; until ricin, the main toxic component, was separated from the main agglutinating component, which was referred to as Ricinus agglutinin [11]. Similar results were later observed with jequirity, and the toxin abrin was isolated from Abrus agglutinin [12].

## 2. The Revival of Interest in Plant Toxins

A quantum leap in the study of RIPs occurred in 1970 when a report of increased in vitro toxicity of abrin and ricin for tumour cells compared with normal cells suggested the possibility of exploiting these toxins for the treatment of cancer [13]. Abrin strongly inhibited protein synthesis in neoplastic cells, while having a moderate inhibitory effect on DNA synthesis and no effect on RNA synthesis [14]. The same results were observed with ricin. Moreover, the inhibition of protein synthesis by either ricin or abrin in tumour cells was faster than in rat liver cells [15].

Olsnes and Pihl at the Institute for Cancer Research in Oslo demonstrated the inhibition of protein synthesis by ricin in a cell-free system in which the elongation arrest of the nascent peptide-chain was observed [16]. These researchers clarified that both ricin and abrin have a heterodimeric structure consisting of two polypeptides: an A chain with toxic activity and a B chain with the properties of a galactose-specific lectin linked by a disulphide bond [17,18].

Various other research groups, in particular researchers at the University of Bologna, were intrigued by ricin’s mechanism of action, which was recognized based on the enzymatic inactivation of ribosomes [17,18,19]. The target of ricin was determined to be the large 60 S subunit of eukaryotic ribosomes [20] and their elongation-factor-dependent GTPase activity [21,22]. In particular, ricin action was shown to target the ribosomal core-particles obtained after removing a number of ribosomal proteins that are involved in the elongation process [23].

In 1987, Endo and colleagues at the Yamanashi Medical College finally discovered the enzymatic activity of ricin [24]. This toxin was identified to be an rRNA N-glycosylase [EC 3.2.2.22] that is able to detach an adenine residue (A4324 in rat 28 S RNA) from a highly conserved ribosomal RNA single-stranded loop that is involved in the binding of elongation factors. The same loop is also the target of alfa-sarcin, another ribotoxin with rRNA endonuclease activity [EC 3.1.27.10] from *Aspergillus giganteus* [25]. Enzymatic activity identical to that of ricin has been detected in various other toxins from plants, including mistletoe lectin I [26] and Vero toxin from bacteria [27]. The same research group also identified a lyase that specifically cleaves the apurinated site of RNA [28]. The wider enzymatic activity of RIPs was ascertained by Barbieri from the Bologna group in 1994. Many of these RIPs are able to deadenylate not only rRNA but also different polynucleotide substrates such as DNA, poly(A) and various types of RNA, including mRNA, tRNA, bacterial rRNA and viral RNA [29,30]. For this reason, the name adenine polynucleotide glycosylase was proposed for RIPs. In addition, it was observed that many RIPs are able to cleave more than one adenine. Thus, the ability to act on various substrates and extensively depurinate some of them suggests that protein synthesis inhibition could be just one of the ways used by RIPs to kill cells.

## 3. The Discovery of New Ribosome-Inactivating Proteins

The golden age of RIP study had already started, and the investigations began to develop in different directions. One of these lines of investigation, mostly developed by Stirpe and colleagues in Bologna, involved the research of new plant proteins with the ability to inhibit protein synthesis using the same mechanism as ricin. Plants known for their toxicity were investigated, and crotin and curcin were isolated from *Croton tiglium* and *Jatropha curcas*, respectively, in 1976 [31]. Surprisingly, these two RIPs had inhibitory effects on cell-free protein synthesis that were similar to the A chain of ricin but lacked the B lectin chain and cell toxicity. A number of other proteins similar to the A chain of ricin were detected in seed extracts in a screening study [32]. Additionally, the highly toxic lectins modeccin from *Adenia digitata* [33,34], viscumin from *Viscum album* [35] and volkensin from *Adenia volkensii* [36] were identified as RIPs with characteristics that were similar to those of abrin and ricin.

In the 1970s, the pokeweed anti-viral protein (PAP) from *Phytolacca americana* was recognized as a single-chain RIP for its ribosome-inactivating activity by Irvin and colleagues at the University of Texas, Austin [37,38,39]. Thus, anti-viral activity became a clue for the discovery of new RIPs, as in the case of dianthin purified from *Dianthus caryophyllus* ([40], reviewed in [41]). Starting in 1988, the evaluation of abortifacient activity became another way to identify new RIPs, as a group of these plant proteins had previously been used for this purpose in traditional Chinese medicine. These proteins include trichosanthin from *Trichosanthes kirilowii* and momorcharins from *Momordica charantia* and were mostly studied by Yeung and colleagues at the Chinese University of Hong Kong ([42], reviewed in [43]).

All of these proteins exhibit ribosome-inactivating activity, and before the enzymatic activity was clarified, this gave rise to their name: RIPs [44]. At that point, it was clear that the name encompassed at least two different categories of proteins: type 1 RIPs consisting of a single polypeptide chain able to inhibit protein synthesis and type 2 RIPs consisting of an A chain with inhibitory activity and a B chain with lectin properties.

For a while, the presence of the B chain was considered sufficient for granting high toxicity. However, some exceptions were already known, such as Ricinus and Abrus agglutinins, which are both type 2 RIPs (reviewed in [45]). In the 1990s, various type 2 RIPs with low toxicity were identified, mostly from Sambucus, by Girbés and colleagues at the University of Valladolid (reviewed in [46]).

Based on an updated list of plant RIPs, the following statements appear clear: (i) they are broadly distributed among different plant genera; (ii) they are expressed by a variety of different tissues and in different isoforms; and (iii) most of them are single-chain proteins (reviewed in [47]).

## 4. Studies of the Ribosome-Inactivating Protein Structure

Ricin was the first RIP to be analysed by X-ray diffraction in 1987 by Robertus and colleagues from the University of Texas, Austin [48]. The ricin A chain (RTA) was found to be a globular protein folded into three domains with a number of α-helical and β-strand structures. The ricin B chain (RTB) consists of two homologous domains, each containing a lactose-binding site and several areas of amino acid homology that were possibly derived from a gene duplication [48,49]. Recently, a comparison of the amino acid sequences revealed similarity between the type 1 RIPs and RTA. However, the complete primary sequence homologies may vary from 15% to 80%. Despite the variability of the primary sequence, the superimposition of the three-dimensional structures of various crystallized type 1 RIPs and RTA have revealed that they share a common fold [50]. In addition, various similarities in the amino acid sequences have been reported among the B chains of type 2 RIPs, especially in the key structural residues that guide the arrangement of the two domains in symmetric structures regardless of the sequence symmetry [51].

Although the three-dimensional structures of ten type 1 RIPs and the A chains of five type 2 RIPs have many similarities, the N- and C-termini can vary in length and therefore assume different conformations. However, a limited number of amino acid residues are highly conserved, and they seem to be important for the catalytic activity because they are directly involved in binding to the substrate or implicated in the mechanism responsible for N-glycosylase activity ([52], reviewed in [53]). In particular, Tyr80 seems to work as a moving door due to its flexibility, which allows the substrate to enter and assume the best favourable orientation in the active site ([54], reviewed in [50]). Other highly conserved amino acids, which have been identified outside of the active site, probably support the activity of RIPs by stabilizing their catalytic conformations (reviewed in [55]).

Many similarities have been reported in the structures of the B chains of different type 2 RIPs, which were studied together with other lectins by Peumans and Van Damme at the Catholic University of Leuven. Nevertheless, some of the differences in the primary sequences have been reported to be crucial for RIP-cell interactions when the galactose-binding residues are involved, thus modulating RIP cytotoxicity [56]. Other highly conserved residues located in the core of the two domains are not involved in sugar binding, but they may be involved in the cytotoxicity of type 2 RIPs by helping to maintain the symmetry of the domains [51].

## 5. Studies of the Genetics and Phylogenesis of Ribosome-Inactivating Proteins

As far as gene structure is concerned, ricin is the best-characterized RIP. In 1985, genomic and transcriptomic analyses by Lord and colleagues at the University of Warwick revealed that ricin is synthesized from a single mRNA that encodes a precursor polypeptide known as pre-pro-ricin. Pre-pro-ricin contains the A and B chain sequences that are separated by a short linker sequence and an N-terminal extension corresponding to the signal peptide that allows for translocation of the protein through the endoplasmic reticulum (ER) [57]. The ricin precursor is glycosylated in the ER lumen and then transferred through the Golgi complex to storage vacuoles [58]. The linker peptide between the two chain sequences is a signal for the vacuolar sorting [59] and is proteolytically removed in the storage vacuoles; thus the precursor protein is processed to mature ricin [60]. During the same period, the complete sequences of some type 1 RIPs were identified [61,62]. Most of the type 1 RIPs, with the exception of those from Poaceae, are synthesized from intron-less genes, which encode precursors with N-terminal and C-terminal extensions. They are synthesized on the ER and then follow the secretory pathway to their final destination to be secreted from the cell or targeted to the vacuoles (reviewed in [63]).

Many plants can produce multiple isoforms of RIP, as in the case of *Viscum album* L. [64], *Phytolacca americana* L. [65,66,67], *Saponaria officinalis* L. [68], *Sambucus ebulus* L. [69], *Mirabilis jalapa* L. [70,71] and *Phytolacca dioca* L. [72,73]. In *Sambucus nigra,* these isoforms exhibit organ-specific and temporal production and are derived either from multi-gene families or from processing and glycosylation of the primary sequence [74].

Sequence analyses have shown that RIPs are widely distributed, even if not ubiquitous, in the plant kingdom (reviewed in [75]). Knowledge concerning the genomic sequences of an increasing number of RIPs has allowed for phylogenetic analysis of their evolutionary history.

Regarding type 2 RIPs, the B chain from the monocot *Iris hollandica* has higher similarity to those from the dicots *Ricinus communis* and *Abrus precatorius,* rather than that from *Polygonatum multiflorum*, the only other known monocot type 2 RIP [56]. In addition, the type 1 RIPs from *Iris hollandica* have a higher sequence similarity to type 2 RIPs from the same species than type 1 RIPs from other monocot species [76]. These observations suggest that all RIPs might be derived from a common ancestor gene and that type 1 RIPs probably originated from the deletion of the B chain-encoding sequence [77].

Since RIPs of the same taxon are often strictly related, it has been hypothesised by the Valladolid group that they evolved in parallel with the differentiation of their respective plants (reviewed in [78]). The Leuven group modified the above hypothesis by considering the presence of RIPs in bacteria. They could have provided the lectin domain for the fusion with an ancestral RIP domain developed in flowering plants 300 million years ago, thus giving rise to type 2 RIPs. With regard to type 1 RIPs, they could have originated independently through a convergent evolutionary process either from the ancestral gene or from the deletion of the lectin domain (reviewed in [75]).

A third hypothesis was later formulated considering that RIP-encoding sequences are also found in fungi and metazoa. The new model suggested that RIP genes could be found in a common ancestor of bacteria, archaea and eukaryotes, and several RIP genes had already begun to evolve before the divergence of the three kingdoms of life [77].

A more recent phylogenetic analysis has shown that all of the angiosperm RIPs share a common ancestor that is similar to the type 1 RIPs of the modern monocots and evolved to form the dicot type 1 RIPs. The merging of a type 1 RIP gene with a duplicated lectin gene formed the type 2 RIPs. In some sort of reverse evolution, the deletion of the lectin domain or the A-chain of the type 2 RIPs gave rise to other type 1 RIPs and lectins, respectively [55].

## 6. Endocytosis and Intracellular Trafficking

Starting in the seventies, researchers began to investigate the interactions of RIPs with cells. The work was mostly done by the Norwegian group and led to the theory of a two-step model for the entry of type 2 RIPs into cells. In the first step, the B chains of abrin and ricin bind to the galactose-containing receptors on the cell membrane. In the second step, the A chain reaches the ribosomes and arrests protein synthesis [79]. HeLa cells have 1–3 × 10^7^ binding sites for abrin and ricin, but only a fraction of them mediate the process of endocytosis of RIPs [80]. Calculations have revealed that a single A chain molecule of either abrin, modeccin or ricin is sufficient to kill one cell after reaching its intracellular target [81].

A large portion of the knowledge regarding RIP-cell interactions has been obtained from ricin. Ricin uptake occurs by receptor-mediated endocytosis in MCF-7 cells, a human breast cancer cell line, through both coated and uncoated pits, as revealed by electron microscopy. Internalization brings the toxin to the endosomal system that leads to both lysosomes and Golgi cisterns [82]. The translocation of ricin to the cytosol does not require the normal function of lysosomes. However, the transport of the toxin to Golgi elements appears to be necessary for intoxication [83], although only 5% of internalized ricin reaches the trans-Golgi network [84]. Accordingly, ricin is not toxic to hybridoma cells that produce anti-ricin antibodies, indicating that only the small fraction of the endocytosed ricin that reaches the secretory pathway is responsible for cell death [85].

From the Golgi apparatus, ricin undergoes retrograde transport towards the ER [86] where the disulphide bond that links the two chains is reduced, possibly by enzymes such as disulphide oxidoreductases [87,88]. Once freed from the B chain, the A chain is translocated to the cytosol following the pathway of misfolded proteins that are directed for degradation in the proteasome through the quality control system referred to as ER-associated protein degradation (ERAD) [89]. A small portion of the ricin A chains can escape degradation by avoiding ubiquitination, probably due to low lysine content [90]. Ricin intracellular trafficking has recently been reviewed, and the discussions involving its translocation to the cytosol emphasized the ER-Golgi cycling of both ricin holotoxin and the free A chain. Above all, the interactions with proteasomal chaperones could allow for a fraction of RTA to be folded into an active conformation instead of being directed to degradation (reviewed in [91]).

The discovery of the retrograde neuronal transport of ricin in 1980 has opened up a large area of research in neurobiology [92], mostly performed by Wiley at Vanderbilt University in Memphis, Tennessee. Similar to ricin and abrin, modeccin [93] and volkensin [94] may also be transported along neuronal processes, and this property has been exploited for the destruction of neuron bodies. The Adeniae toxins modeccin, volkensin [95] and stenodactylin [96] can be retrogradely transported not only in peripheral nerves but also in the rat central nervous system. Neuronal suicide transport allows for the selective lesioning of neurons by injecting the toxin into their projection area (reviewed in [97]).

The low toxicity of some type 2 RIPs was investigated by the Bologna group, as well as by Robertus and colleagues. It may depend on (i) a scarce number of binding sites on the cell surface for the lectin B chain, as in the case of IRA from *Iris hollandica* [98] or (ii) a high degradation due to different intracellular routing that avoids accumulation in the Golgi apparatus, as observed with nigrin B from *Sambucus nigra* L. [99,100]. Likewise, the low toxicity of ebulin from *Sambucus ebulus* L. and Ricinus agglutinin, compared with ricin, to cultured human T cells has been ascribed to variations in cell binding, uptake, or membrane translocation [101].

Less information is available regarding the internalization of type 1 RIPs. They do not possess the B chain and are predominantly taken up by pinocytosis. Gelonin is internalized by lymphoid cells through the formation of pinosomes [102]. In macrophages, gelonin may follow two internalization pathways: a mannose receptor-mediated endocytosis, or a fluid-phase pinocytosis that appears to be the more relevant route for intoxication [103].

PAP shows the same range of cytotoxicity to cultured human T cells as the low-toxicity lectins ebulin and Ricinus agglutinin, and it is internalized by fluid-phase uptake in a manner similar to the isolated A chain of ricin [95]. In yeast cells, PAP is able to be retro-translocated from the ER into the cytosol and escape proteasomal degradation similar to the A chain of type 2 RIPs [104].

Endocytosis via LDL receptor family members has been reported for RTA and saporin in the human monocyte-like U937 cell line [105], although the sensitivity of different cell lines to saporin is not correlated to the expression levels of the α-2-macroglobulin receptor [106]. Receptor-mediated endocytosis of trichosanthin has also been described in the choriocarcinoma JAR cell line, which is highly sensitive to RIPs, but not in the less sensitive hepatoma H35 cell line [107]. Indeed, JAR cells showed a high uptake of trichosanthin possibly mediated by their high level of LDL receptor-related protein 1 [108]. In addition, trichosanthin might undergo pH-dependent membrane insertion that has been attributed to the last seven C-terminal residues and could be essential for trichosanthin translocation into the cytosol (reviewed by [109]).

Receptor-independent endocytosis of saporin in HeLa cells has been described and is followed by the distribution of the RIPs in the endosomal compartment, accumulation in perinuclear vesicles and nuclear localization. Moreover, saporin intoxication induces the formation of DNA gaps due to the presence of nuclear abasic sites [110].

## 7. Cytotoxicity and Toxic and Adverse Effects in Animals and Humans

The toxicity to animals of different RIPs is highly variable and depends on the ability of reaching the intracellular targets. Indeed the cytotoxicity is strictly related to the toxic effects to the organism (reviewed in [111]).

Different cell types show variable levels of sensitivity to RIP cytotoxicity. For instance, macrophagic [112] and trophoblastic cells [113] exhibit variable levels of sensitivity, possibly because the former is equipped with mannose receptors and the latter is very active in pinocytosis, both of which may facilitate endocytosis. The ability of RIPs to induce cell death through an apoptotic mechanism was first suggested [114] and then demonstrated [115,116]. In addition, the ability of RIPs to stimulate various cell death mechanisms makes them appear more attractive for the treatment of cancer than conventional chemotherapy, in which one of the major problems is the rise of resistant cells [117,118]. 

The low toxicity of type 1 RIPs justifies the absence of documented cases of accidental poisoning of animals or humans. The intravenous administration of α-momorcharin at high doses may induce toxic symptoms in rats including lethargy, shortness of breath, blood in the stool and convulsions leading to death. Moreover, chronically treated animals develop inflammatory cell infiltration and spotty necrosis in the liver [119]. Injection of saporin causes necrosis of the kidney tubule epithelium in mice because of the reabsorbtion of filtered saporin by the renal tubules [120]. Mice receiving lethal doses of bryodin, gelonin, momordin, PAP, saporin, trichokirin and momorcochin-S exhibit severe necrotic liver damage [121]. Intraperitoneal injection of trichosanthin at sub-lethal doses causes the death of proximal tubule cells in rats, thus inducing renal failure. In this case, cell death is possibly due to reabsorption of the filtered trichosanthin and has the features of both apoptosis and necrosis [122].

The only spontaneous pathology induced by type 1 RIPs is the onset of allergies. Indeed, work-related sensitization and the induction of IgE production have been reported for the Bologna researchers, upon exposure to dianthin, gelonin, momordin, PAP and saporin, as well as ricin and volkensin [123]. Indeed, the allergenic activity of castor bean has long been known because of occupational sensitization of workers in industries that produce ricinus oil, as well as in the population living in the neighbourhood [124,125]. Trichosanthin has been used for medical purposes as both an abortifacient [126,127] and an anti-viral drug. Major adverse effects were observed in phase II clinical trials for the treatment of HIV, and the AIDS patients developed neurological disorders, flu-like symptoms and anaphylactic reactions [128,129].

Similar to type 1 RIPs, no documented cases of fortuitous poisoning of animals or humans have been reported for low-toxicity type 2 RIPs. However, allergic reactions to elderberry have been reported, which are possibly related to RIPs [130].

The high toxicity of toxic type 2 RIPs to animals and humans has been well known since the earliest times, and lesions and pathological manifestations caused by intoxication with these plant lectins have been reported (reviewed in [111,131]). In particular, the effects of ricin poisoning have recently been described after the accidental ingestion of castor bean or as a result of voluntary actions related to suicide, homicide or even terroristic purposes (reviewed in [132]). Abrin intoxication is rarer. However, the consequences of jequirity bean intake have been described in detail (reviewed in [133]) and have been reported in a case of attempted suicide [134]. The incidental consumption of *Adenia digitata* fruits has been reported to cause fatal poisoning in humans [135]. Although no profound toxicity has been associated with the accidental ingestion of American mistletoe berries [136], a case of human hepatitis was attributed to the consumption of an herbal remedy containing mistletoe ([137], reviewed in [138]). Additionally, anaphylactic reactions have been reported after therapeutic injection of mistletoe [139].

## 8. Applications in Medicine as Immunotoxins

In recent years, the potential uses for plant toxins as drugs have become clear. Although they possess highly efficient cell-killing mechanisms, plant toxins lack selectivity towards cell targets. To overcome this problem, some researchers have explored the possibility of linking them to carriers that are specific for targets on unwanted cells. The most widely used carriers are antibodies, and the corresponding conjugates are referred to as “immunotoxins” (ITs). The first IT was generated in 1976 when Moolten and colleagues at the University School of Medicine in Boston linked RTA to the anti-(C58NT)D globulin [140]. Two strategies were initially adopted to construct ITs. In the first one, the antibody was linked to the entire type 2 toxin. In the second one, the antibody was linked to the A chain alone or to a type 1 RIP. The first type 2 RIPs used to construct ITs were ricin, as a whole molecule [141] or as an A-chain [140,142,143,144], and abrin [145]. Despite the high efficiency of the ITs constructed with whole toxins, their highly nonspecific toxicity hampered their use, and it became necessary to block, modify or delete the lectin chain (reviewed in [146]).

To date, more than a hundred different ITs have been constructed with ricin, many fewer with abrin (less than a dozen) and very few ITs have been constructed with other type 2 RIPs (reviewed in [147]). Since the 1980’s, most ricin-containing ITs have been prepared using the A chain alone. The process involves the reduction of the disulphide bond and further purification (RTA), eventually followed by chemical deglycosylation (deglycosylated A chain, dgRTA). Many of these studies were performed by Vitetta’s group at the University of Texas Southwestern Medical Center in Dallas starting in 1982 [148] and by Frankel’s group starting in 1987 at Duke University [149].

In addition to type 2 RIPs, many type 1 RIPs have also been employed to construct ITs, and several hundreds of different ITs have been produced. Thorpe and colleagues at the Chester Beatty Research Institute of London constructed the first IT containing the type 1 RIP gelonin [150]. Later, ITs against leukaemia cells were prepared with PAP by Huston and colleagues at the University of Kansas, Lawrence [151] and with gelonin by Lambert and colleagues at the Dana-Farber Cancer Institute in Boston [152]. However, saporin-S6 has been the most widely used type 1 RIP for the construction of ITs (reviewed in [153,154]), mainly due to the thermodynamic stability of its molecular structure [155,156,157]. The first saporin-containing ITs were constructed by Thorpe and colleagues in 1985, and they coupled the toxin to a monoclonal anti-Thy 1.1 antibody and its F(ab’)2 fragment [158]. At that time, the anti-tumour effects of saporin-containing ITs were being exploited by other research groups [120,159]. In several cases, the type 1 RIP-containing ITs were more active than those containing RTA [160,161].

ITs have been exploited for the elimination of many pathologic cells responsible for various illnesses, but the best results have been obtained in the haematological field involving both tumour therapy and immunological disorders. Since the 1980’s, many clinical trials have been conducted involving RIP-containing ITs. The first of such trials based on the anti-CD5 RTA-T101 IT showed encouraging results after being conducted on two leukaemia patients who were refractory to chemotherapy [162]. Thereafter, many other clinical trials were performed using RIP-containing ITs to treat several different neoplastic diseases (reviewed in [147,163]). IT-based immunotherapy often produced better results than traditional chemotherapy ([164], reviewed in [165]).

Despite the successful results reported in clinical trials of ITs, several problems were encountered including poor penetration into solid tumours. To overcome this problem and obtain a more standardized drug formulation, a second generation of ITs were conceived. They were constructed using recombinant hybrid molecules in which the carrier (usually variable antibody fragments) and the RIP were fused using recombinant DNA techniques to obtain conjugates of smaller sizes to facilitate diffusion into tumours (reviewed in [166]).

ITs have also been used for the prevention and control of transplant rejection and GvHD. In the early 1980’s, different ricin-containing ITs directed against antigens on mature T lymphocytes were evaluated by Vallera and colleagues at the University of Minnesota, Minneapolis, and both the in vitro and in vivo studies produced very promising results [167,168].

ITs have also been used in ex vivo bone marrow purging in autologous transplantation. The ex vivo treatments had the advantages of avoiding the side effects caused by the injection of ITs into patients and allowing for the possibility of using ITs that cross-react with other tissues. Pioneering studies in this direction were performed by Vitetta’s research group using RTA-containing ITs against the human lymphoblastoid B-cell line [169]. The first ITs to be used for ex vivo bone marrow purging were obtained by conjugating antibodies against lymphocyte antigens to the type 2 RIP ricin in 1986 [170] and to the type 1 RIP momordin in 1989 [171].

ITs have also been applied to the treatment of autoimmune diseases, and the first studies on this topic date back to the 1990’s (reviewed in [172]). Anti-CD5/RTA was the first IT to be used in clinical trials for the treatment of many autoimmune diseases, including rheumatoid arthritis, systemic lupus erythematosus and insulin-dependent diabetes mellitus [173,174,175,176].

In the 1990’s, ITs were also exploited for the treatment of AIDS with the goal of eliminating the HIV-infected T cells that release the active virus and spread the infection. Two of the main approaches were as follows: (i) ITs recognizing the viral antigens expressed on the surface of infected cells (i.e., gp120 and gp41) and (ii) ITs directed against antigens on CD4+ lymphocytes, the cells in which the virus replicates (reviewed in [177]). All of the ITs conceived for HIV treatment were constructed using the type 1 RIP PAP. However, RIP-containing ITs have never entered clinical trials for the treatment of AIDS patients (reviewed in [178]).

Another therapeutic application for RIP-containing ITs is in nanosurgery for the treatment of oculo-facial dystonia or eye movement disorders, which provides an alternative to botulinum toxin [179]. Additionally, saporin-S6 containing ITs (conjugated to substance P, anti-NGFR antibodies, etc.) have been widely used in neuroscience studies to selectively destroy neurons. These ITs represent important models for behavioural studies, studies of Alzheimer’s disease and other neuronal loss pathologies, and for the therapy of some forms of strong chronic pain (reviewed in [180]).

## 9. Biological Roles and Future Perspectives

A defensive role has been attributed to toxic type 2 RIPs, and they are thought to defend the plant producing them by intoxicating predator animals that are sensitive to the ingestion of their seeds or tissues. The antiviral activity of type 1 RIPs confirms their defensive role ([38,181], reviewed in [41]). In addition, these plant enzymes exhibit antibacterial [182,183], antifungal [184,185,186], anti-protozoan [187] and insecticidal [188] properties (reviewed in [189]).

The defence against foreign pathogenic invaders may depend on the ability of RIPs to elicit an apoptotic response in the infected cells, thus limiting the spread of the pathogen. Otherwise, the RIPs protective actions may involve direct action against pathogens. For instance, various type 1 RIPs are toxic to the larvae of lepidopteran insects and cause them to develop extensive DNA lesions [190].

The mechanical wounding of *Phytolacca insularis* leaves is able to induce the expression of the antiviral protein PIP2. The increase of this RIP in response to mechanical stress may be a protective mechanism against pathogen and pest attacks, which are facilitated by the mechanical injury of plant tissues [191]. Moreover, variations in the RIP expression of plants in relation to different environmental changes suggests a role for these proteins in the adaptation to both biotic and abiotic stresses by inducing the apoptosis of cells that were destined to die [192,193].

The high concentrations of some RIPs in seeds and underground storage organs provides support for the hypothesis that these proteins play a role in storage. This theory does not contradict the defensive role of RIPs because the abundance of RIPs in storage tissues could both provide them with nutritional benefits and defend against pathogens. For example, dual functions as anti-pathogen and storage proteins have been attributed to ME1 and ME2, two type 1 RIPs isolated from the roots of *Mirabilis expansa*. Indeed, in addition to constituting approximately 20% of root proteins, ME1 and ME2 also exhibit antibacterial and antifungal activities [194].

Plant genetic engineering using RIPs has been applied in agriculture to increase resistance and protect crops from microbial pathogens and pests, while limiting the use of environmentally aggressive chemical methods of disease control [195,196,197].

The intratumour injection of recombinant adeno-associated virus vectors containing the trichosanthin gene was attempted to inhibit the growth of human hepatocellular carcinoma tumours in a murine xenograft model [198]. Despite the promising results reported, further studies are needed to state the validity of this approach.

However, the most promising applications for RIPs in the future are medical and mostly involve the conjugation of RIPs to carrier molecules, particularly antibodies, to build ITs. As outlined above, these conjugates can specifically eliminate the target cell of the antibody and may have many applications, especially in the treatment of cancer. Recently, the use of nanomaterials instead of antibodies has been proposed as a novel therapeutic approach for the biomedical applications of RIP (reviewed in [199]).

**Figure 1:**
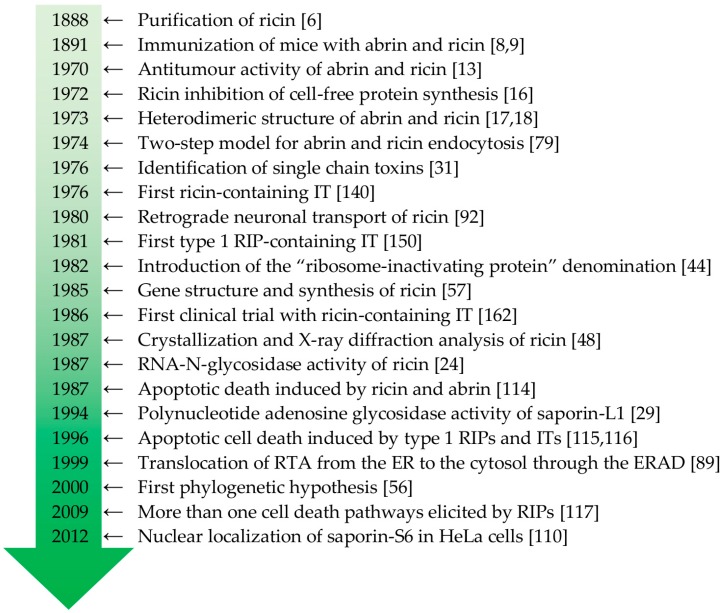
Timeline of milestones in RIP research. ER: endoplasmic reticulum; ERAD: endoplasmic reticulum-associated protein degradation; IT: immunotoxin; RIP: ribosome-inactivating protein; RTA: ricin toxin A-chain.

Over more than a century, numerous studies have been published on RIPs involving a large number of researchers in various fields. The main achievements of the research on this topic are summarized in Figure 1.
